# A collagen orientation switch reshapes fin architecture

**DOI:** 10.1016/j.isci.2026.117464

**Published:** 2026-09-09

**Authors:** Rintaro Tanimoto, Kazuhide Miyamoto, Koji Tamura, Shigeru Kondo, Junpei Kuroda

**Affiliations:** 1 Graduate School of Frontier Bioscience, Osaka University, Suita, Osaka, Japan; 2 Laboratory of Organ Morphogenesis, JT Biohistory Research Hall, Takatsuki, Osaka, Japan; 3 Department of Ecological Developmental Adaptability Life Sciences, Graduate School of Life Sciences, Tohoku University, Sendai, Miyagi, Japan; 4 Graduate School of Science, Osaka University, Toyonaka, Osaka, Japan

**Keywords:** collagen fiber, extracellular matrix, actinotrichia, fin-to-limb transition, collagen imaging

## Abstract

Fibrillar-collagen architecture is a fundamental determinant of vertebrate tissue morphology; yet, its organizational principles remain poorly understood because collagen structures are challenging to visualize *in vivo*. Actinotrichia, the fibrillar-collagen structures in fish fins, provide an ideal system: they are readily visualized and exhibit a simple, tractable spatial pattern optimal for studying collagen organization. Using high-sensitivity 3D imaging, we revealed the essential role of the fish-specific *actinodin* genes in establishing this pattern. Our findings demonstrate that *actinodin1* and *actinodin2* are indispensable for forming the fin’s collagen architecture and for generating its thin, elongated morphology. Loss of *actinodin* function reorients collagen fibers and transforms the fin bud into a limb-bud-like configuration. A similar collagen organization is observed in developing amphibian limbs, which lack *actinodin* genes. These findings suggest that alterations in fibrillar-collagen patterning have contributed to morphological divergence and position *actinodin* mutants as promising models for investigating collagen matrix formation.

## Highlights

• 3D collagen imaging reveals an essential role for *actinodin* in fin morphogenesis
• Actinodin is required for maintaining collagen fibers parallel to the fin epidermis
• *actinodin* KO reorients collagen fibers perpendicular to the fin epidermis
• *actinodin*-KO paired fins acquire a limb-like collagen configuration and morphology

## Graphical abstract

**Figure:**
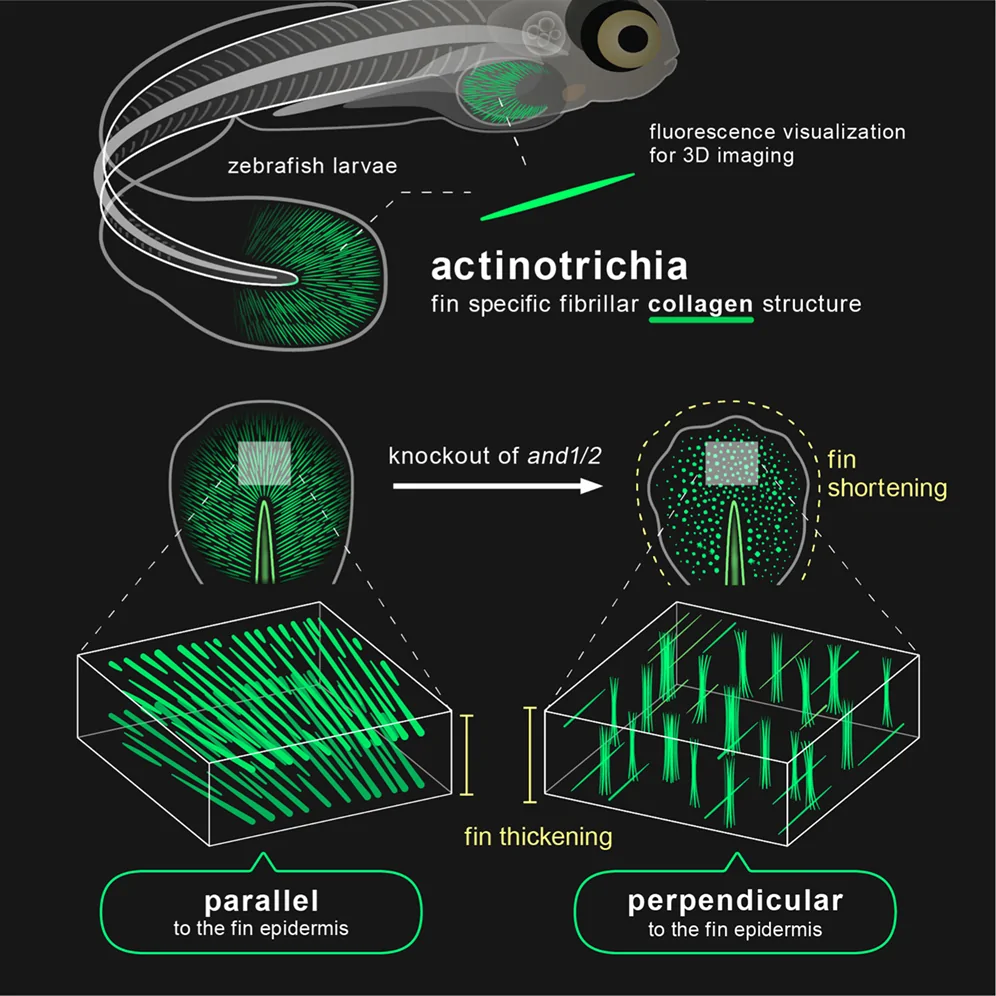

## Teaser

Biological sciences; Cell biology; Developmental biology; Evolutionary biology; Natural sciences

## Introduction

The orientation and distribution of fibrillar collagen are critical determinants of the shape and mechanical properties of bones and organs.^1^^,^^2^^,^^3^ However, how they are spatially organized within vertebrate tissues is still poorly understood,^4^^,^^5^^,^^6^^,^^7^ as visualizing these collagen architectures remains challenging.

Actinotrichia (AT) are large fibrillar-collagen structures located at the distal tips of fish fins.^8^^,^^9^^,^^10^^,^^11^ They play indispensable roles in fin morphogenesis by providing structural support and guiding tissue shaping.^12^^,^^13^^,^^14^^,^^15^ Owing to their considerable size (up to 300 μm in zebrafish), distinctive spear-like morphology, and highly ordered arrangement—forming parallel layers beneath the epidermis with individual fibers extending distally—AT have recently emerged as a powerful model for studying collagen fiber organization.^16^^,^^17^^,^^18^^,^^19^^,^^20^ These features have enabled recent studies to identify cellular^18^^,^^19^^,^^20^ and extracellular matrix (ECM)^12^^,^^13^^,^^15^^,^^21^ factors involved in pattern formation of AT distribution.

Two major cell types—the basal epidermal cells (bECs)^17^^,^^18^^,^^22^ in the basal layer of the fin epidermis and the mesenchymal cells (MCs)^9^^,^^19^^,^^23^ in the fin mesenchyme—play essential roles in AT assembly and patterning. AT are positioned between the mesenchymal tissue and the basement membrane,^9^^,^^19^^,^^24^ suggesting that these cells regulate AT organization. Previous studies indicate that they secrete and interact with AT components, including fibrillar collagens (Col1a1a, Col2a1b),^13^^,^^25^ FACIT collagens (Col9a1c),^15^^,^^21^^,^^26^ and non-collagenous proteins such as Actinodin1 and Actinodin2.^12^^,^^14^^,^^19^ Among these components, actinodin (And) proteins encoded by *actinodin* (*and*) genes are characterized by unique structural features: the gene family contains a unique repeat motif (C[N/D]PXXDPXC), yet its function cannot be inferred from sequence because it shows no homology to any known gene family.^12^ Notably, *and* genes were lost in the tetrapod lineage and have been proposed to contribute to the fin-to-limb transition.^12^^,^^27^^,^^28^^,^^29^^,^^30^^,^^31^^,^^32^^,^^33^ In zebrafish, they are specifically expressed in the distal fin-tip tissues during development, growth, and regeneration, coinciding with the region where AT are distributed.^12^^,^^14^ Knockdown of *actinodin*1 (*and1*) and *actinodin2* (*and2*) disrupts AT production and fin-fold development, and these proteins localize to AT.^12^ Together, these findings implicate *and* genes in collagen fiber assembly as components of AT. Despite these insights, the developmental impact of disrupting *and* genes has not been examined in detail, and their functional roles remain unclear.

In this study, we highlight the specific roles of *and* genes in collagen organization and fin morphogenesis by applying a high-sensitivity 3D collagen imaging technique using diaminofluorescein-FM diacetate (hereafter referred to as DAF in this study) staining to *and1/2* double-knockout (dKO) zebrafish. Our recent work demonstrated that the DAF probe fluorescently labels collagen fibers in whole-mount living tissue by reacting with allysine residues in collagen crosslinking intermediates, thereby forming covalent bonds with collagen molecules.^34^ Although the transient staining may partially interfere with collagen crosslinking, its toxicity is sufficiently low to allow normal AT formation and tissue development.^20^ This approach enables non-destructive *in vivo* visualization of collagen architecture in whole-mount tissue.^20^^,^^34^ Using this method, we show that loss of *and1/2* reorganizes collagen fibers into a limb-like pattern and shifts fin morphology toward a limb-like state.

## Results

### Loss of *and1/2* reorients collagen fibers and alters fin morphology in zebrafish larvae

As described above, a previous report analyzed the function of And1/2 by simultaneously knocking *and1/2* down with morpholinos.^12^ However, this approach might only partially suppress the function of these proteins. Therefore, in this study, we generated a dKO zebrafish line of *and1/2* using CRISPR-Cas9^35^ (Figure S1). To confirm that the *and1* and *and2* KO alleles indeed abolish the expression of functional proteins, we performed whole-mount staining using anti-And1/2 antibodies. In the wild-type fin fold, fibrous signals outlining the AT were clearly detected, consistent with previous studies showing that both And1 and And2 localize to this structure.^12^^,^^14^^,^^18^^,^^19^ In contrast, no such signals were observed in either the *and1* KO or *and2* KO fin fold, indicating that these alleles eliminate the expression of functional proteins (Figure S2).

**Figure 1:**
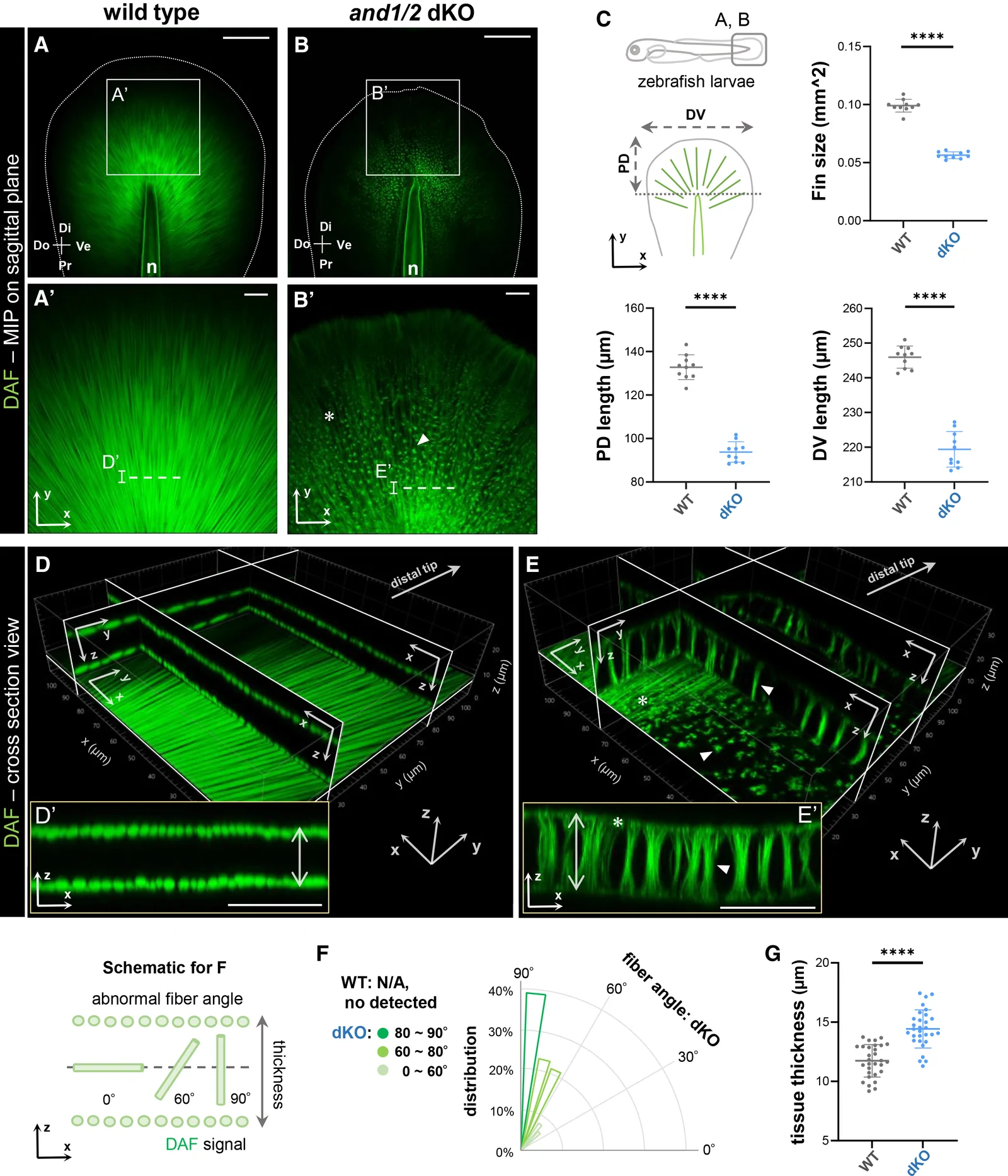
Double knockout of *and1/2* alters the organized alignment of collagen fibers in the caudal fin fold of zebrafish (A–B’) Confocal images of the caudal fin fold in wild-type and *and1/2* dKO zebrafish at 5 days post-fertilization (dpf). Collagen fibers were fluorescently labeled (green) by whole-mount DAF staining. Magnified views of the white-boxed regions are shown in the lower panels. White dotted lines: fin outlines; white asterisk: abnormally thin fibrous structures; white arrowhead: abnormal particle-like structures. Pr: proximal; Di: distal; Do: dorsal; Ve: ventral; n: notochord; MIP: maximum intensity projection. Scale bars: 100 μm (A and B), 20 μm (A’ and B’). (C) Quantification of fin morphology. Data are presented as mean ± SD. Welch’s *t* test: ∗∗∗∗*p* < 0.0001. WT vs. dKO; *N* = 10 per group. (D–E’) Orthogonal slice view from (A’ and B’); MIP, 5-μm stack, the region 50 μm from the notochord end (D’ and E’). White asterisks: abnormally thin fibers; white arrowheads: abnormally oriented fibers; white double-headed arrows: thickness of the interstitial space. Scale bars: 20 μm. (F) Distribution of abnormal fiber angle measured from optical section. WT: N/A; dKO: *N* = 172 (10 larvae per group). (G) Quantification of fin mesenchyme thickness (the region 50 μm from the notochord end). Data are presented as mean ± SD. Welch’s *t* test: ∗∗∗∗*p* < 0.0001. WT vs. dKO; *N* = 30 per group (10 larvae per group).

First, we examined the phenotype of the caudal fin fold because AT is distributed throughout this tissue and can be readily observed.^9^^,^^11^ Specifically, to analyze the relationship between collagen fiber orientation and tissue morphological changes, we performed high-resolution 3D imaging of collagen structure within the fin fold using our previously reported DAF staining method.^20^^,^^34^ Figures 1A and 1B show fluorescence images of the caudal fin fold captured from a lateral view. Compared with the wild-type, the dKO exhibited a reduced fin size, with lengths shortened along both the proximodistal and dorsoventral axes (Figure 1C). Each magnified fluorescence images of the area surrounding the posterior end of the notochord is shown in the panels below (Figures 1A’ and 1B’). In the wild-type, the AT radiated distally from the base of the caudal fin fold. Their thickness were uniform (2–3 μm) and equivalent to that of typical collagen fibers (1–20 μm) found in vertebrate tissues.^36^ In contrast, in the dKO, the overall fluorescence of the fin tissue was reduced, and the radially oriented fibers were thinner than wild-type AT (Figure 1B’, a white asterisk). Furthermore, particularly in the region near the base of the caudal fin fold, strong punctate fluorescence was observed (Figure 1B’, a white arrowhead). To clarify the structural features of this fluorescence signal, we enlarged the relevant region and performed a 3D reconstruction (Figures 1D and 1E). In the wild-type, fibrous structures were aligned immediately beneath the epidermis on both sides, with no collagen-like structures visible in the spaces between them. In contrast, in the dKO, fibers running parallel to the epidermis were present, but these fibers were thinner than wild-type AT (Figure 1E, white asterisks). Moreover, aberrant fiber bundles appeared in the interstitial spaces (Figure 1E, white arrowheads), which were not present in the wild-type. Measurement of the angles of these abnormal fibers relative to the epidermis revealed that their distribution was not random but clearly biased toward a perpendicular orientation (Figure 1F). Abnormal bundle thickness and spacing were largely constant, with maxima of ∼3 μm. Consequently, imaging from a lateral view showed that the signals exhibited a spotted pattern (Figure 1E, white arrowheads). In addition, optical section images revealed that the thickness of the fin interstitial space in the dKO increased by 20% compared with the wild-type (Figures 1D’, 1E’, and 1G).

Next, to determine the contribution of each gene to this phenomenon—the formation of abnormally oriented fibers—we similarly examined the collagen structures in caudal fin fold of each genotype combination. Specifically, we assessed whether abnormal collagen fiber structures were present in the spaces between the normally oriented fibers that run parallel to the fin epidermis, as observed in the dKO (Figure S3). In the *and1 +/+*, *and2 −/−* (Figure S3A) and *and1 −/−*, *and2 +/+* (Figure S3B), almost all fibrous structures ran parallel to the epidermis, similar to the wild type, i.e., no significant changes were observed in the collagen matrix distribution. In the *and1 −/+*, *and2 −/−* (Figure S3C) and *and1 −/−*, *and2 −/+* (Figure S3D), abnormal fibrous structures were observed similar to the dKO (Figures S3C” and S3D”, white arrowheads), but their number was significantly lower compared with the dKO. The relationship between the number of abnormal fibers and each genotype is shown in Figure S3E.

Furthermore, because the phenotype observed in the zebrafish *and1/2* dKO lines was unprecedented, we performed the same observations using DAF staining on the recently reported *and* mutant of rainbowfish^37^ to determine whether this phenomenon is specific to zebrafish or shared among fish. As shown in Figures S4A and S4B, we confirmed the presence of vertical collagen structures around the base of the caudal fin fold of the mutant, similar to those observed in zebrafish, whereas such structures were absent in the wild-type.

Thus, *and1* and *and2* are presumed to share the same function in collagen orientation, namely directing collagen fibers to align parallel to the fin epidermis, which promotes distal fin elongation. Conversely, in the absence of *and* genes, these collagen fibers shift to a perpendicular orientation relative to the fin epidermis, potentiate resulting in suppression of distal outgrowth and proximal tissue expansion.

### A shift in collagen orientation reorganizes mesenchymal cell layers

**Figure 2:**
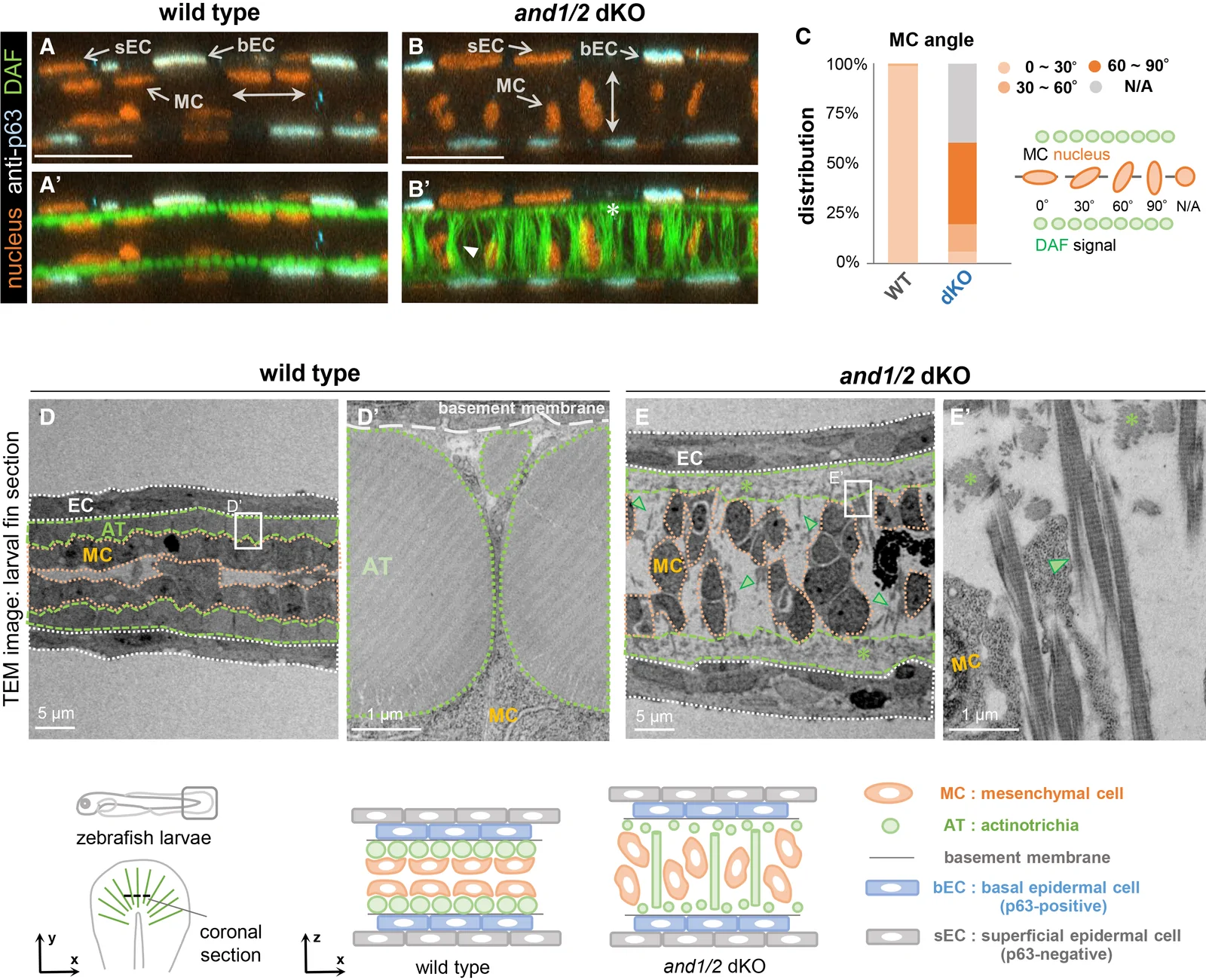
Abnormal MC elongation and collagen fiber orientation in the caudal fin fold of *and1/2* dKO zebrafish (A–B’) Representative optical sections from wild-type and *and1/2* dKO zebrafish at 5 dpf (MIP, 5-μm stack, the region 50 μm from the notochord end). Collagen fibers were fluorescently labeled (green) by whole-mount DAF staining, all cell nuclei were stained with Hoechst (orange), and bEC nuclei were labeled with anti-p63 antibodies (light blue). White asterisk: parallel-oriented fibers; white arrowhead: perpendicular-oriented fibers. MC: mesenchymal cell nucleus; bEC: basal epidermal cell nucleus (p63-positive); sEC: superficial epidermal cell nucleus (p63-negative). Scale bars: 20 μm. (C) Distribution of MC nuclear orientation relative to the sagittal line. WT: *N* = 92; dKO: *N* = 106 (9 larvae per group). (D–E’) TEM images for the coronal section of caudal fin fold in wild-type and *and1/2* dKO zebrafish at 8 dpf. Green asterisks: parallel collagen fibers; green arrowheads: perpendicular collagen fibers. Schematic diagram of larval fin tissue anatomy is shown in the lower panel.

Next, since the orientation of collagen fibers is likely determined by the surrounding cells that express *and1/2*, we examined the state of these cells by fluorescently labeling their nuclei—bECs (p63-positive) in the basal epithelial layer,^12^^,^^18^^,^^22^^,^^24^ which give rise to superficial epidermal cells (sECs; p63-negative) in the outer epithelial layer, and MCs in the fin mesenchyme (Figures 2A–2C).^9^^,^^12^^,^^19^^,^^23^ Figures 2A and 2B show optical section images of a coronal cross-section of the caudal fin fold, acquired and visualized in a manner comparable to Figure 1D’ and 1E’. As in the wild type, p63-positive bECs in the *and1/2* dKO were detected exclusively on the outer side of the collagen structures. No obvious differences were observed in the distribution of epidermal cell nuclei (bECs and sECs) between the wild-type and the dKO. Epidermal cells in both genotypes exhibited a flattened morphology along with the fin plane. In contrast, significant changes were observed in the distribution and morphology of the nuclei of MCs. In the wild-type, the MCs were located immediately inside of the AT (Figure 2A’). However, in the dKO, the MCs were positioned between the vertical fibers (Figure 2B’, a white arrowhead), rather than immediately beneath the fibers distributed on the basal side of the epidermis (Figure 2B’, a white asterisk). The angular distribution of the major axis of MCs (approximated as ellipses) relative to the epidermis is shown in Figure 2C. This analysis confirms that in the wild-type, almost all MCs were flattened along the AT, whereas in the dKO, they tended to be oriented perpendicular to the epidermis.

Additionally, to examine in detail abnormalities in collagen structures and alterations in cell distribution, we performed comparative observations of caudal fin fold sections using transmission electron microscopy (TEM) (Figures 2D and 2E). In the wild-type, the epidermal cells (ECs: bECs and sECs), the AT, and the MCs were arranged in distinct layers from the outer to the inner side of the tissue, forming a stacked structure (Figure 2D). High-magnification imaging of the boundary region between these three layers revealed that the AT, approximately 2–3 μm in diameter, were orderly aligned immediately beneath the ECs. Characteristic crystalline structures previously reported for AT were clearly observed within them^9^^,^^19^^,^^21^^,^^24^ (Figure 2D’). The MCs formed a layer on the inner side of the AT, adhering to their surface, while the mesenchyme further inward was extremely thin. On the other hand, in the dKO, although the overall layered organization of the tissue was comparable to that of the wild-type, the mesenchyme displayed a clearly different appearance (Figure 2E). In the region where the AT is present in the wild-type, collagen structures with diameters of several hundred nanometers were scattered and aligned parallel to the epidermal layer (Figures 2E and 2E’, green asterisks). The average width of their distribution was approximately 3 μm, consistent with the width of the AT layer in the wild-type. Moreover, the mesenchyme further inward from this region was clearly thicker than that in the wild-type. The MCs were scattered throughout this area, and surrounding them were vertically oriented collagen bundles, each several hundred nm thick (Figures 2E and 2E’, green arrowheads). A characteristic striped pattern was visible within these structures, consistent with the typical 67-nm pitch of collagen fibers.^1^^,^^36^ These observations supported the findings from confocal imaging using DAF staining.

Together, these results suggest that loss of And function prevents MCs from elongating along the proximal-distal axis beneath the epidermis and instead causes them to move into the deeper mesenchyme, where they produce collagens oriented perpendicular to the epidermis. This shift in MC behavior leads to a reorientation of collagen fibers from a parallel to a perpendicular alignment relative to the fin epidermis. Consequently, while distal elongation of the larval fin is suppressed, tissue thickness is inferred to increase.

### Alterations in collagen orientation and fin morphology in the *and1/2* dKO persist even after bone formation

**Figure 3:**
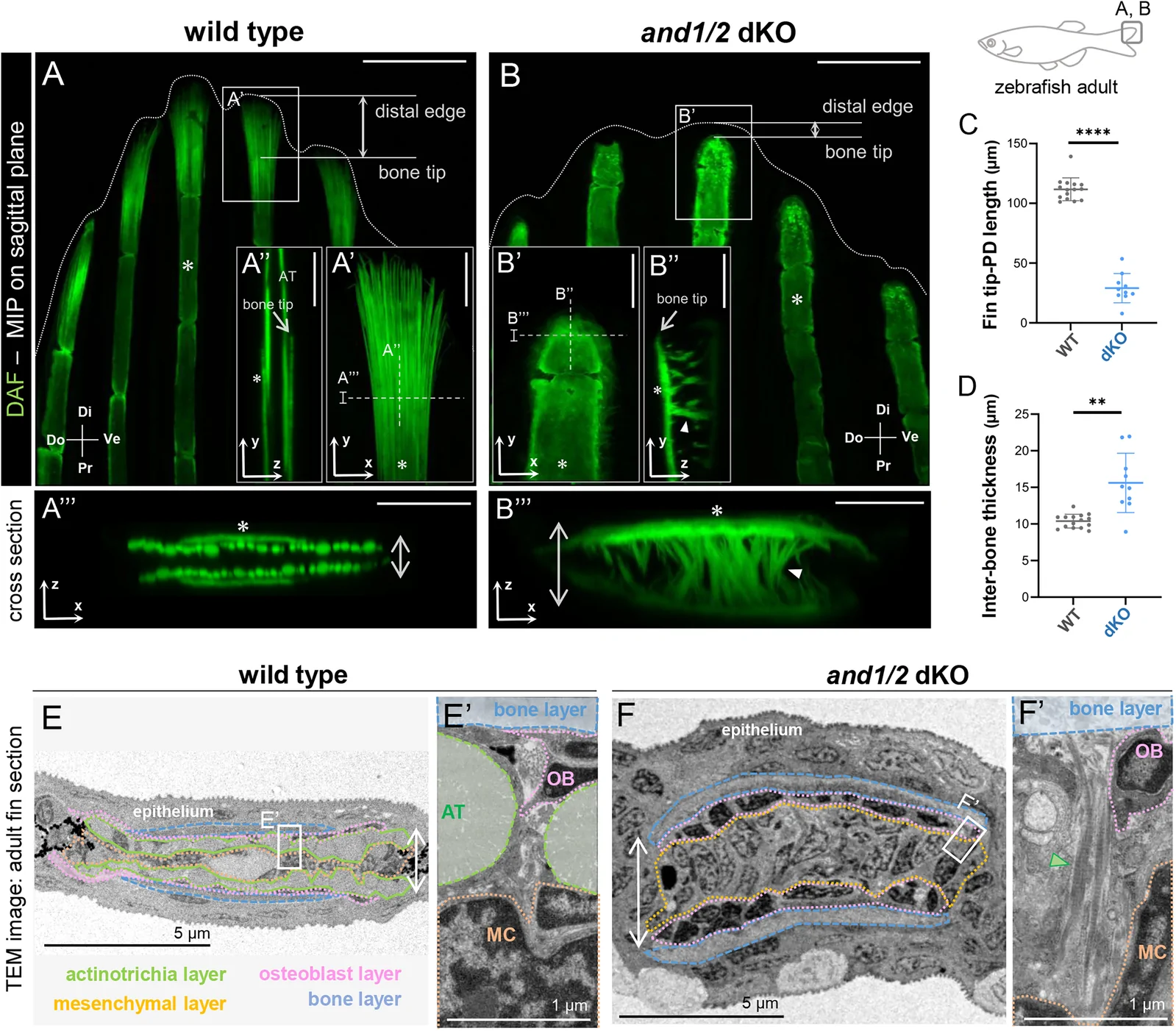
Abnormal collagen fiber distribution in the caudal fin of *and1/2* dKO zebrafish (A–B’’’) Confocal images of the caudal fin tip in wild-type and *and1/2* dKO zebrafish with standard length (SL) = 2.1 ± 0.1 cm (adults over 6 months of age). Collagen fibers were fluorescently labeled (green) by whole-mount DAF staining. The magnified images in the white box are shown as insets. Cross-sectional views at the white dotted lines are shown in the side panels (A’’ and B’’) and in the lower panels (MIP, 10-μm stack; A’’’ and B’’’). White dotted lines: fin outlines; white asterisks: collagen matrices within fin bones; white arrowheads: abnormally oriented collagen structures; white double-headed arrows: thickness between left and right fin bones. AT: actinotrichia. Scale bars: 200 μm (A and B), 50 μm (A' and B'), 20 μm (A’’–A’’’ and B’’–B’’’). (C and D) Quantification of fin bone morphology. Data are presented as mean ± SD. Welch’s *t* test: ∗∗*p* < 0.01, ∗∗∗∗*p* < 0.0001. WT: *N* = 14; dKO: *N* = 10 (10 fishes per group). (E–F’) TEM images for the coronal section of caudal fin tip in wild-type and *and1/2* dKO zebrafish with SL = 2.1 ± 0.1 cm (adults over 6 months of age). Green arrowhead: abnormally oriented collagen fiber; white double-headed arrows: thickness between left and right fin bones. AT: actinotrichia; MC: mesenchymal cell; OB: osteoblast.

During the larval stage described above, AT are present throughout the fin. By contrast, in adults, fin rigidity is provided by fin bones, and AT are restricted to the growing tips of these bones.^8^^,^^10^^,^^13^^,^^14^ To determine whether collagen orientation changes occur under this condition, we next performed a detailed analysis of the caudal fin phenotype in the *and1/2* dKO line. Accordingly, to test whether the collagen abnormalities seen in larvae also occur at the fin tips in adults, we performed DAF staining and examined their distribution (Figures 3A and 3B). Figures 3A and 3B show fluorescence images of the fin tip tissue imaged from a lateral view. In the wild-type, the fin bones connected by segments extended linearly along the distal axis, forming regularly spaced rectangular units (Figure 3A, a white asterisk). Furthermore, strong fibrous fluorescence signals aligned along this axis were detected in the terminal ∼200 μm region of each fin bone. As shown in the magnified image, the AT were densely distributed at the bone tip (Figure 3A’). In cross-sectional views of the bone tip (Figures 3A” and 3A”’), the AT were arranged parallel to the inner surface of the bone. However, in the dKO, the rectangular fin-bone units were distorted, and no fibrous structures were observed at the bone tip (Figure 3B’). The distance from the fin-bone tip to the distal edge of the fin tissue along the proximodistal axis was significantly reduced (Figures 3A–3C). Remarkably, cross-sectional observation of the distal tip region of the fin bone clarified abundant collagen structures running perpendicular to the bone (Figures 3B” and 3B”’). In addition, the mesenchymal tissue filling the space between the left and right fin bones were markedly thickened (Figures 3A’’’, 3B’’’, and 3D). Hence, the fin tip tissue of the adult also exhibited changes in collagen orientation and tissue morphology, similar to those observed in the larval stage.

These alterations were further supported by TEM observations, as shown in Figures 3E and 3F. Specifically, in the wild-type, the AT were observed immediately inside the fin bone (Figures 3E and 3E’, blue region), but in the dKO, no AT-like structures were detected in this region. Instead, collagen fibers arranged perpendicular to the fin bones were observed, forming a bundled pattern similar to that seen in the larval fin (Figure 3F’, a green arrowhead). The mesenchymal tissue between the left and right fin bone layers was also markedly thicker than that in the wild-type (Figures 3E and 3F). Consistently, this phenomenon—collagen fibers oriented vertically relative to the fin bones due to *and* deficiency—was also observed in rainbowfish after ossification (Figures S4C and S4D).

As shown in Figure 3, the dKO zebrafish also exhibited abnormalities in the fin bones, which form the skeletal framework of fins. Therefore, we also analyzed morphological changes in the caudal fin (Figure S5). This analysis confirmed that the dKO caused fin shortening, abnormal ray patterning, and misshaping of bone segments (Figure S5). The same morphological changes were also observed in the pectoral fins (Figure S6), consistent with recent studies.^30^^,^^38^ Moreover, as in the caudal fin, a similar alteration in collagen distribution was confirmed (Figure S6). Aside from the fin phenotype, adult *and1/2* KO fish showed no apparent defects in overall morphology, development, growth, or viability (data not shown).

These findings indicate that, *and* deficiency induces a common set of phenotypes—altered collagen orientation, local tissue thickening, and distal suppression of fin elongation—which arise regardless of life stage or fin type and are broadly conserved among fish.

### Perpendicular collagen-fiber organization is shared by *and*-deficient fins and limbs

**Figure 4:**
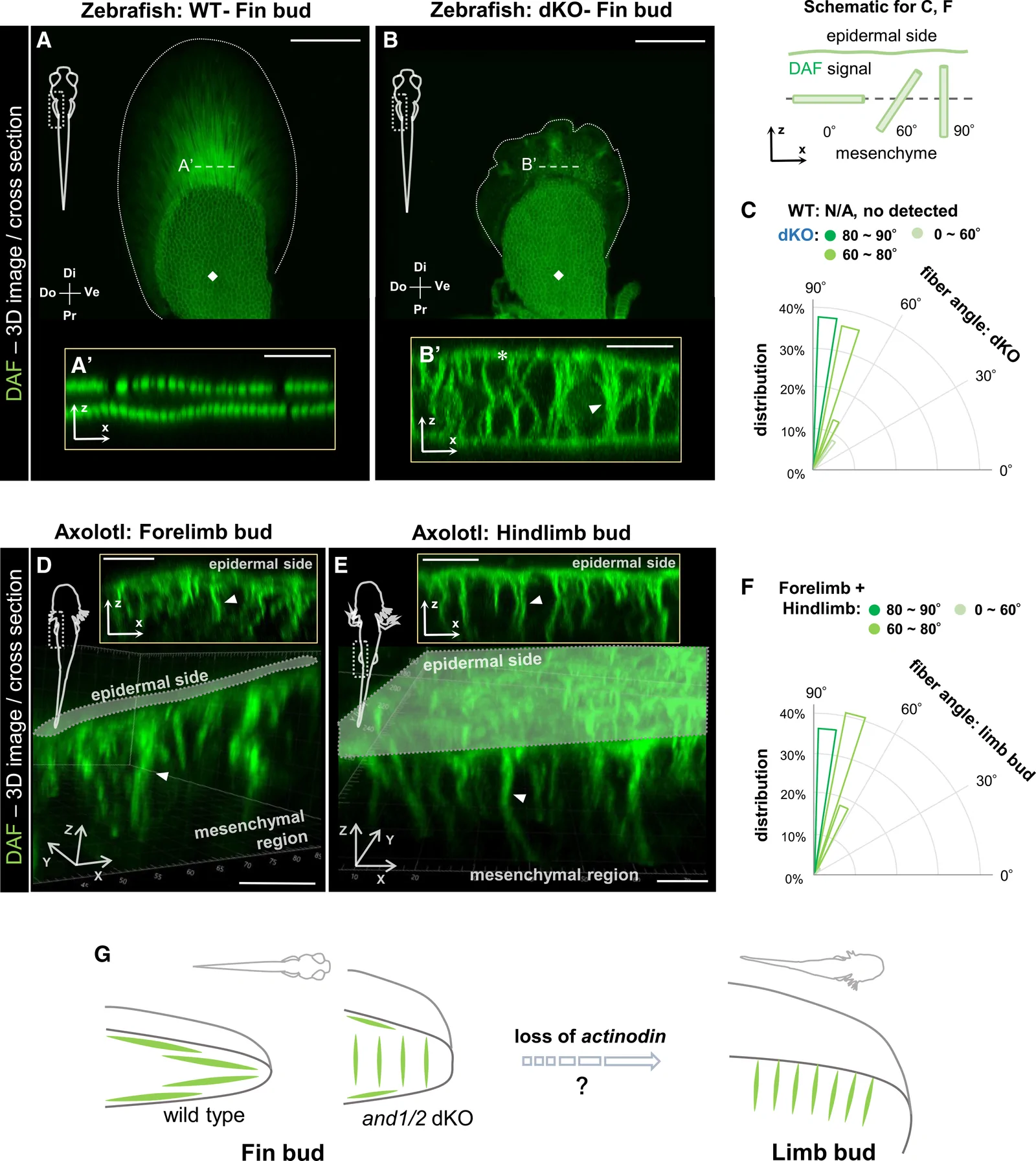
Aberrant collagen fiber orientation arises in the pectoral fin buds of *and1/2* dKO zebrafish, comparable to that observed in axolotl limb buds (A–B’) Confocal images of the pectoral fin bud in wild-type and *and1/2* dKO zebrafish at 5 dpf; MIP, 5-μm stack, the region 10 μm distal to the endoskeletal disc edge (A’ and B’). White dotted lines: fin outlines, white diamonds: endoskeletal discs, white asterisk: parallel-oriented fiber, white arrowhead: vertically oriented fiber. Scale bars: 100 μm (A and B), 10 μm (A’ and B’). (C) Distribution of abnormal fiber angle in pectoral fin bud measured from optical section (the region 10 μm distal to the endoskeletal disc edge). WT: N/A (8 larvae); dKO: *N* = 121 (4 larvae). (D and E) Confocal images of limb bud in axolotl (MIP, 20-μm stack [cross section]). White arrowheads: collagen fibers perpendicular to the epidermal layer. Scale bars: 10 μm. (F) Distribution of fiber angle in limb bud measured from optical section. *N* = 336. (G) Schematic diagram summarizing the key insight of this study.

As mentioned above, functional loss of the fish-specific proteins And1/2 caused collagen fibers in the fin to align perpendicular to the epidermis, accompanied by tissue thickening along the direction of collagen orientation. If this finding has evolutionary significance,^12^ collagen fibers should tend to adopt a perpendicular orientation beneath the epidermis of the organ that corresponds to the fin in vertebrates lacking the *and* gene family—that is, in tetrapods. Thus, we examined the distribution of collagen fibers beneath the epidermis in limb buds of amphibians—the tetrapod lineage most closely related to fish^39^—and in the homologous structure,^40^ the paired fin bud, using the same method applied to the caudal fin fold of zebrafish. Figures 4A and 4B show fluorescence images of the collagen fiber organization in the pectoral fin buds of zebrafish. In the wild-type, similar to the caudal fin fold, the AT were neatly aligned only in a direction parallel to the epidermis. In contrast, in the dKO, in addition to collagen fibers aligned in the same direction (Figure 4B’, a white asterisk), aberrant collagen fibers were formed and extended across the mesenchyme (Figure 4B’, a white arrowhead). As shown in Figure 4C, the angular distribution of these structures revealed a distinct bias toward a perpendicular orientation relative to the epidermis. Moreover, fin bud shortening and increased thickness of the mesenchyme surrounding the endoskeletal disc were also observed (Figures 4A’ and 4B’).

Finally, to examine collagen-fiber formation during limb development—the organ homologous to the paired fins—we analyzed fluorescent 3D images obtained from axolotl limb buds using the same method (Figures 4D and 4E). In both forelimb and hindlimb buds, collagen fibers extending into the mesenchyme were observed (Figures 4D and 4E, white arrowheads). Notably, the orientation distribution was indistinguishable from that observed in the *and1/2* dKO fin buds, with both showing approximately 80% of fibers oriented between 70° and 90° (Figures 4C and 4F). Hence, we found that collagen fibers oriented perpendicular to the limb-bud epidermis were present in amphibians, which innately lack the *and* gene family.

These findings demonstrate that, in the absence of both And1/2, subepidermal collagen fibers in fin buds and limb buds adopt a perpendicular orientation to the epidermis.

## Discussion

In this report, we revealed that the fish-specific gene *and1/2*^12^ is essential for establishing the ordered alignment of collagen fibers in fin tissue and for forming an elongated, thin fin structure. Analysis using high-resolution 3D imaging demonstrated that *and1/2* deficiency induces abnormal vertical alignment of collagen fibers, resulting in fin-bud shortening and thickening, and transition to a limb-like morphology. We further found that collagen fibers with a similar vertical orientation are also formed in the limb buds of amphibians, a tetrapod group most closely related to fish. Our findings suggest that the emergence of this collagen-fiber orientation may represent one of the factors that facilitated the evolutionary transition from fins to limbs.

In fish fins, AT are known to align parallel to the epidermal layer,^8^^,^^9^ and previous studies suggested that And1/2 are involved in AT production as AT are composed of these ECM proteins.^12^^,^^14^^,^^19^ By generating *and1/2* dKO fish and applying a highly sensitive collagen-fiber imaging method we recently developed,^20^^,^^34^ we uncovered a previously unrecognized role for And1/2: beyond AT formation, And1/2 prevent the vertical alignment of collagen fibers and thereby promote fin-tissue thinning. These proteins presumably couple the dynamics of bECs and MCs by mediating ECM formation, thereby promoting the arrangement of collagen fibers in a parallel orientation beneath the basement membrane.

This mechanism likely arises from processes occurring during early fin development. First, local sheets of epidermal cells extend outward to form the initial ridge structure, known as the apical ectodermal ridge. These derived bECs secrete And proteins and fibrillar collagens to the subepidermal space, thereby initiating AT formation beneath the basement membrane in a parallel orientation.^22^^,^^24^ Second, MCs migrate distally from the proximal region, guided along the inner side of the AT within the subepidermal space.^9^^,^^41^ At this step, MCs likely recognize the bEC-derived AT as a scaffold, which in turn drives their elongation parallel to the epidermis in a manner similar to durotaxis.^19^^,^^41^^,^^42^ Finally, bECs and MCs collaboratively produce And proteins and fibrillar collagens to drive the distal AT growth, leading to the formation of a thin, distally elongated fin-bud structure known as the apical fold.^12^^,^^43^ Thus, And1/2 are required for proper fin outgrowth by directing the organization of collagen fibers to align parallel to the fin epidermis.

A future challenge is to elucidate the molecular mechanism by which And1/2 regulate collagen-fiber orientation. How these proteins interact with other factors remains unknown, both in the context of ECM formation (e.g., collagen,^13^ fibronectin,^44^ fibulin,^45^ fibrillin,^46^ and hemicentin^45^^,^^46^) and in the regulation of MC dynamics (e.g., integrins,^47^ BMP,^48^ and sphingosine^49^). Clarifying these interactions is expected to provide a molecular and cellular understanding of how the unique collagen architecture of fin tissue is generated.

In addition to the structural alterations described here, the abnormal collagen fiber organization observed in the *and1/2* dKO likely reflects changes in molecular pathways involved in collagen modification and remodeling. Enzymes such as P4H, PLOD2, LOX family members,^36^^,^^50^ and MMPs^51^ are known to regulate collagen hydroxylation, cross-linking, and turnover. Alterations in their expression or activity could contribute to the formation of orthogonally oriented or irregular collagen bundles in the mutants. Although the present study focused on the structural consequences of And1/2 loss, future analyses of these molecular markers will be essential for elucidating the biochemical mechanisms underlying the phenotype.

Furthermore, our findings offer new insight into the evolutionary significance of the loss of the *and* gene family.^12^^,^^27^^,^^28^^,^^29^^,^^30^^,^^31^^,^^32^^,^^33^ The loss of *and* genes may have facilitated the vertical alignment of collagen fibers in the ancestors of tetrapods, contributing to the fin-to-limb transition. The fin-bud formation process described above suggests the following model. First, loss of *actinodin* function suppresses the initial AT formation by bECs, resulting in much thinner collagen fibers parallel to the fin epidermis. Second, although MCs migrate distally from the proximal region into the subepidermal space, the initially formed parallel fibers provide insufficient scaffold cues for MC recognition, causing them to relocate into the deeper mesenchymal region. Finally, these MCs form collagen fibers around themselves within the deeper mesenchymal region, leading to fibers that extend across the mesenchyme and are oriented perpendicular to the fin epidermis. This sequence may trigger subsequent changes in cellular dynamics, ECM distribution, and ultimately tissue morphology, thereby generating a limb-bud-like architecture.

More broadly, this work highlights ECM organization as a potential driver of morphological evolution, complementing conventional models centered on signaling molecules and transcription factors.^52^^,^^53^^,^^54^^,^^55^^,^^56^ The ECM is increasingly recognized not merely as a supportive scaffold but as a dynamic regulator of cell proliferation, differentiation, and migration.^57^^,^^58^^,^^59^ In both fins and limbs, fibrillar collagen formation is initiated during the elongation phase of bud development,^60^^,^^61^^,^^62^^,^^63^^,^^64^ suggesting that changes in collagen-fiber orientation can be decisive factors in morphogenesis. Collectively, our findings support a model in which loss of And1/2 reorganizes collagen-fiber architecture and mesenchymal-cell dynamics, thereby converting fin buds into a limb-bud-like configuration.

Overall, this study identifies collagen-fiber reorganization as a previously underappreciated factor contributing to the evolutionary divergence between thin fins and thickened limbs. The identification of mutants that dramatically alter collagen-fiber orientation provides a valuable tool for uncovering the mechanisms underlying collagen-matrix assembly.^65^^,^^66^^,^^67^^,^^68^^,^^69^^,^^70^^,^^71^

### Limitations of the study

Although this study demonstrates that loss of *and1/2* reorganizes collagen fibers and alters fin morphology, our interpretation that MC dynamics are altered in the *and1/2* dKO is based on nuclear staining and TEM observations, which do not capture full cell morphology. More detailed analyses using cell-type-specific reporters will be required to determine how cell shape, cytoskeletal architecture, and cell-matrix adhesion structures are altered in the *and1/2* dKO. In addition, the functional relationship between And proteins and collagen molecules is still unclear. Whether And1 and And2 act redundantly or possess distinct biochemical activities has not been directly tested in this study. Subtle phenotypic differences among *and1/2* single- and double-mutant combinations could reflect differences in gene expression rather than protein function. Future comparative interactome analyses will be necessary to identify potential binding partners and to clarify how And proteins contribute to collagen-matrix assembly.

## Resource availability

### Lead contact

Requests for further information and resources should be directed to and will be fulfilled by the lead contact, Junpei Kuroda (junpei.kuroda@brh.co.jp).

### Materials availability

Zebrafish lines generated in this study are available from the lead contact with a completed materials transfer agreement.

### Data and code availability

Data reported in this study will be shared by the lead contact upon request. This study does not report original code. Any additional information required to reanalyze the data reported in this study are available from the lead contact upon request.

## Acknowledgments

We thank Midori Tanaka and Rie Taniguchi, technical staff members at Kondo Laboratory, Graduate School of Frontier Biosciences, Osaka University, for fish maintenance. We also thank Dr. Ritsuko Suyama, Dr. Yasuhiro Hirano, Dr. Ryosuke Kaneko, and Dr. Ritsuko Morita for managing the confocal microscopes at the Graduate School of Frontier Biosciences, Osaka University. We acknowledge the Leica imaging Lab at Osaka University for the support in obtaining fluorescence imaging data. This research was funded by 10.13039/501100001691JSPS KAKENHI, grant number 20H05943, 10.13039/501100001691JSPS KAKENHI, grant number 21K06200, 10.13039/501100001691JSPS KAKENHI, grant number 23H04301, JST FOREST Program, grant number JPMJFR224P, and JST SPRING, grant number JPMJSP2138.

## Author contributions

Conceptualization, J.K.; methodology, R.T. and J.K.; investigation, R.T., K.M., and J.K.; writing – original draft, R.T. and J.K.; writing – review and editing, R.T., K.M., K.T., S.K., and J.K.; visualization, R.T. and J.K.; supervision, J.K.; project administration, J.K.; funding acquisition, R.T., S.K., K.T., and J.K.

## Declaration of interests

The authors declare no competing interests.

## Declaration of generative AI and AI-assisted technologies in the writing process

During the preparation of this work, the authors used Copilot and DeepL to assist with English editing. After using this tool or service, the authors reviewed and edited the content as needed and take full responsibility for the content of the publication.

## STAR★Methods

### Key resources table

**Table:** 

REAGENT or RESOURCE	SOURCE	IDENTIFIER
**Antibodies**		

Mouse monoclonal anti-p63 (clone BC4A4)	Abcam	Cat#ab735; RRID: AB_305870
Rabbit polyclonal anti-And1 peptide	Cosmo Bio	Custom-made; Antigen: RGYGGRYPGQDDH
Rabbit polyclonal anti-And2 peptide	Cosmo Bio	Custom-made; Antigen: PVPRFEDFLRAYMGGYK
F(ab’)2-Goat anti-Mouse IgG (H + L) Cross-Adsorbed Secondary Antibody, Alexa Fluor™ 594	Thermo Fisher Scientific	Cat#A-11020; RRID: AB_2534087
Alpaca anti-Rabbit IgG Nano (VHH) Recombinant Secondary Antibody, Alexa Fluor™ 568	Thermo Fisher Scientific	Cat#SA5-10325; RRID: AB_2868372

**Chemicals, peptides, and recombinant proteins**		

EnGen® Spy Cas9 NLS	NEB	Cat#M0646
Tricaine (MS-222)	Sigma-Aldrich	A5040; CAS: 886–86-2
DAF-FM DA	Goryo Chemical	Cat#SK1004-01
Hoechst 33342 (Cellstain®)	Dojindo	Cat#H342
Alizarin Red S	Sigma-Aldrich	Cat#A5533

**Critical commercial assays**		

KOD FX *neo*	TOYOBO	Cat#KFX-201
BigDye™ Terminator v3.1 Cycle Sequencing Kit	Thermo Fisher Scientific	Cat#4337456
Hi-Di™ Formamide	Thermo Fisher Scientific	Cat#4311320

**Experimental models: Organisms/strains**		

Zebrafish: AB strain (wild type)	Zebrafish International Resource Center (ZIRC)	ZFIN: ZDB-GENO-960809-7
Zebrafish: *and1/and2* double-knockout line	This paper	N/A
Rainbowfish (Melanotaenia praecox): wild type	Miyamoto et al.^37^	N/A
Rainbowfish (Melanotaenia praecox): *and1/and2* mutant	Miyamoto et al.^37^	N/A
Axolotl (Ambystoma mexicanum)	Amphibian Research Center, Hiroshima University	N/A

**Oligonucleotides**		

gRNA: *and1*_gRNA: AGGGTCACAAGACTTGACTGGGG	This paper	N/A
gRNA: *and2*_gRNA: AGGCAGTAAGGGTCAAGGTAAGG	This paper	N/A
Primer: *and1*_Forward: CTCTGGCTGGTGGTACCTTG	This paper	N/A
Primer: *and1*_Reverse: TTGCAGAGCTGGATCGTGTC	This paper	N/A
Primer: *and1*_Sequence: ACAGATGCGCAGCAGCTCAG	This paper	N/A
Primer: *and2*_Forward: ACAGGGCCATCTGACCAATC	This paper	N/A
Primer: *and2*_Reverse: TAGCAGTCATAACCTTCCTTGG	This paper	N/A
Primer: *and2*_Sequence: CCACAGATTGAGGACTTGGATCG	This paper	N/A

**Software and algorithms**		

ApE (A Plasmid Editor)	M. Wayne Davis	RRID:SCR_014266; https://jorgensen.biology.utah.edu/wayned/ape/
ZEN software (blue edition, version 3.5)	Carl Zeiss	https://www.zeiss.com/microscopy
Imaris (version 10.1.1)	Oxford Instruments	https://imaris.oxinst.com
Fiji (ImageJ)	NIH	https://fiji.sc
GraphPad Prism (version 10)	GraphPad Software	https://www.graphpad.com

### Experimental model and study participant details

#### Animal maintenance

The AB strain was used as the wild-type zebrafish in all experiments. Zebrafish were maintained under standard conditions at 28 °C with a 14 h light/10 h dark cycle. Axolotls were maintained at 20 °C under the same 14 h light/10 h dark photoperiod. Rainbowfish (*Melanotaenia praecox*) were maintained as previously described.^37^ Animals were anesthetized with tricaine (MS-222) at concentrations adjusted according to body size. Zebrafish and axolotl experiments were approved by the Animal Care and Use Committee of Osaka University (permit numbers FBS-14–002-1 and FBS-22–010) and JT Biohistory Research Hall (permit number 43-1). Rainbowfish experiments were approved by the Tohoku University Animal Research Committee (permit number 2022LsA-002-10).

### Method details

#### Establishment of *and1/and2* double knockout (*and1/2* dKO) zebrafish line

To generate founder fish for the *and1/2* dKO line, guide RNAs (gRNAs) targeting *and1* and *and2* were designed. The gRNAs and Cas9 protein were co-injected into one-cell–stage fertilized eggs of wild-type AB zebrafish, producing the F0 mosaic generation. Individuals in the F0 population exhibiting abnormal fin morphology were selected and intercrossed to obtain the F1 generation, which also displayed fin abnormalities. F1 individuals were crossed with wild-type fish, and their offspring were genotyped (see Methods section below). Fish carrying knockout alleles for both *and1* and *and2* (Figures S1A and S1B) were selected as the F2 generation (*and1* −/+, *and2* −/+). Intercrossing the F2 population yielded the *actinodin1/actinodin2* double-knockout (*and1/2* dKO) zebrafish line.

#### Genotyping of zebrafish

For DNA extraction, whole embryos or half bodies of larvae were collected, and caudal or anal fin samples were collected from adult fish anesthetized with 0.02% tricaine in tank water. Samples were incubated in 25–50 μL of DNA extraction buffer (10 mM Tris-HCl, 2 mM EDTA, 0.2% Triton X-100, and 200 μg/mL Proteinase K in Milli-Q water) at 56 °C for 2 h to overnight. Proteinase K was inactivated at 70 °C for 10 min, and the resulting DNA solution was used as the template for PCR with KOD FX *neo*. PCR products were purified by ethanol precipitation and subjected to sequencing reactions using BigDye Terminator. The primers used for genotyping are listed in the table. PCR reaction compositions and cycling conditions followed the manufacturer’s instructions. After ethanol precipitation, the dried pellets were dissolved in Hi-Di Formamide, and sequencing was performed using a 3500 Genetic Analyzer (Applied Biosystems). Sequence alignment was conducted using ApE (A Plasmid Editor) with reference sequences obtained from the NCBI database and the UCSC Genome Browser.

#### DAF staining and tissue fixation for zebrafish

DAF-FM DA (DAF) staining was performed under the same conditions for all samples, regardless of subsequent experimental procedures. Living fish were incubated in 5 μM DAF solution overnight (500 μM stock in DMSO diluted 1:1000 in tank water). The following morning, fish were washed in fresh tank water for approximately 1 h. After anesthesia in 0.02% tricaine, samples (whole bodies or fin tissues) were collected, rinsed in ice-cold PBS, and fixed in 4% PFA in PBS overnight. Fixed samples were stored in PBS at 4 °C until further use. For samples not subjected to additional staining, fixed tissues were directly mounted and imaged using a confocal microscope (LSM780, Carl Zeiss) equipped with Plan Apo 10×/0.45 and C-Apochromat 40×/1.20 W objectives (Carl Zeiss). DAF fluorescence was excited and detected using a 488 nm laser line.

#### DAF staining for the visualization of collagen fibers in rainbowfish fin

For whole-mount staining of rainbowfish (M. praecox), DAF staining and fixation were performed using the same procedures as for zebrafish. For fluorescence imaging, fixed samples were mounted on glass-bottom dishes, and fin tissues were imaged using a confocal microscope (Stellaris8, Leica) equipped with 20× NA 0.8 Plan Apo objectives (Leica). Fluorescent signals from DAF-labeled collagen fibers were excited and detected using a 488 nm laser line.

#### DAF staining for the visualization of collagen fibers in axolotl tissues

For whole-mount staining, living axolotl larvae at stages 47 (forelimb) and stages 54 (hindlimb) were incubated in 5 μM DAF solution (500 μM stock in DMSO was diluted in breeding water, 1: 1000) under dark conditions for 12 h at 20 °C. After staining, they were anesthetized with tricaine at an optimal concentration and fixed with 4% PFA in PBS overnight at 4°C. After the fixation, their limbs were dissected and observed with a confocal microscope (LSM780, Carl Zeiss) equipped with 20× NA 0.8 Plan Apo objective (Carl Zeiss). For the observation using a confocal microscope, the fluorescent signals of the collagen fibers stained with DAF were detected using a 488 nm laser.

#### Antibody and nuclear staining of fin tissues

For antibody staining, fixed samples (DAF-stained at 4–5 dpf) were washed in 0.2% PBS-T at room temperature (RT; 10 min × 3), briefly immersed in ethanol for solvent exchange, and then immersed in acetone at −30 °C for 20 min. Afterward, samples were washed in 0.2% PBS-T at RT (10 min × 3) and transferred to blocking buffer (1 mg/mL BSA in PBS-T), followed by gentle shaking for 1 h at RT. Subsequently, samples were incubated in each primary antibody solution at 4 °C overnight (anti-p63, anti-And1, or anti-And2; 1:100 in blocking buffer). The following day, samples were washed in 0.2% PBS-T at RT (10 min × 3) and transferred to secondary antibody solution at each condition as follows. For anti-p63: Goat anti-Mouse IgG, Alexa Fluor™ 594 (1:200 in blocking buffer, mixed with Hoechst at 1:100), incubated at RT for 2 h. For anti-And1/2: Alpaca anti-Rabbit IgG, Alexa Fluor™ 568 (1:500 in blocking buffer), incubated at RT for 1 h. After that, samples were washed in 0.2% PBS-T at RT (10 min × 3) and then rinsed with PBS, and stored at 4 °C until imaging. For fluorescence imaging, samples were mounted on glass-bottom dishes, and fin tissues were imaged using a confocal microscope (LSM780, Carl Zeiss) equipped with 20× NA 0.8 Plan Apo objective and C-Apochromat 40×/1.20 W objectives (Carl Zeiss). Each fluorescence signal was excited with a 561 nm laser line and detected through the corresponding emission channel.

#### Bone staining of adult zebrafish fins using Alizarin Red S

Before the experiments, the standard length (SL) of adult fish over 6 months of age in each group was measured, and individuals with an SL of 2.1 ± 0.1 cm were selected for subsequent analyses. For Alizarin Red staining, living fish were incubated in 0.0005% Alizarin Red S solution (diluted in tank water) overnight. The following day, fish were washed in fresh tank water for approximately 1 h. After anesthesia with 0.02% tricaine, whole fish were mounted in a glass dish, and fluorescence images of the caudal fins were acquired using a BZ-X810 microscope (KEYENCE) with an mCherry filter.

#### TEM imaging for zebrafish fin tissue section

Fin tissues from larval (8dpf) and adult (with SL = 2.1 ± 0.1 cm, over 6 months of age) zebrafish were fixed overnight at 4°C in 2% glutaraldehyde (GA) and 2% paraformaldehyde (PFA) prepared in 0.1 M cacodylate buffer. Samples were post-fixed in 2% osmium tetroxide (OsO4) in 0.1 M cacodylate buffer for 2 h at 4°C, stained with 2% uranyl acetate in distilled water for 15 min at room temperature, and subsequently stained with lead solution for 3 min at room temperature. The specimens were dehydrated through a graded ethanol series (50%, 70%, 90%, and 100%), embedded in Quetol-812 epoxy resin, and polymerized at 68°C for 48 h. Ultrathin sections (70 nm) were cut using a Leica UCT ultramicrotome and examined with a JEOL JEM-1400 Plus transmission electron microscope operated at 100 kV.

### Quantification and statistical analysis

#### Image analysis and statistical analysis

Imaging data were viewed and analyzed using ZEN software, Imaris, and ImageJ. Maximum-intensity projections and cross-sectional images were visualized using ZEN software (Figures 1, 2, 3, 4, S2, S3, and S6). 3D reconstruction images were acquired using Imaris (Figures 1, 4, and S4). Fin tissue morphology (Figures 1, 3, and S5), collagen orientation and distribution (Figures 1, 4, and S3), and mesenchymal cell nuclear angle distribution (Figure 2) were quantified using Fiji (ImageJ) with the Straight Line and Angle tools.

For zebrafish larvae, cross-sectional regions of interest (ROIs) were cropped from the 3D imaging data as maximum-intensity projections (MIPs; X: 100-μm width; Z: 5-μm stack). For the caudal fin fold, the ROI was positioned 50 μm from the end of the notochord, and for the pectoral fin bud, 10 μm distal to the edge of the endoskeletal disc. For Figures 1F and 4C, the DAF signals running parallel to the epidermis on both sides of the fin were approximated as straight reference lines, and the angles of DAF-positive collagen fiber structures crossing the space between these lines were quantified. Using the same criteria, the number of such abnormal DAF-positive fiber structures was counted for Figure S3E. Similarly, for Figure 2C, the angles of mesenchymal cell nuclei were quantified by approximating each nucleus as an ellipse and measuring the orientation of its long axis.

For the axolotl limb bud, ROIs were cropped from the 3D imaging data as MIPs covering the full imaging field in X and a 20-μm z stack. For Figure 4F, DAF signals located beneath and along the epidermis were used as reference lines, and the angles of DAF-positive fiber structures extending through the mesenchyme were quantified.

Statistical analyses were performed using GraphPad Prism. All plotted data are shown as mean ± SD. For Figure S3E, multiple comparisons were conducted using the Tukey–Kramer method. Except for Figure S3E, each comparison test was performed using Welch’s *t* test. All *p* values <0.05 were considered statistically significant.
