# Supplementary material for: A collagen orientation switch reshapes fin architecture
Source: iScience. 2026 Sep 9;29(10):117464. doi: 10.1016/j.isci.2026.117464 (PMC13573005; doi:10.1016/j.isci.2026.117464)
Supplement: Supplementary file 1 [file mmc1.pdf]

iScience, Volume *29*

Supplemental information

A collagen orientation switch reshapes fin architecture

Rintaro Tanimoto, Kazuhide Miyamoto, Koji Tamura, Shigeru Kondo, and Junpei Kuroda

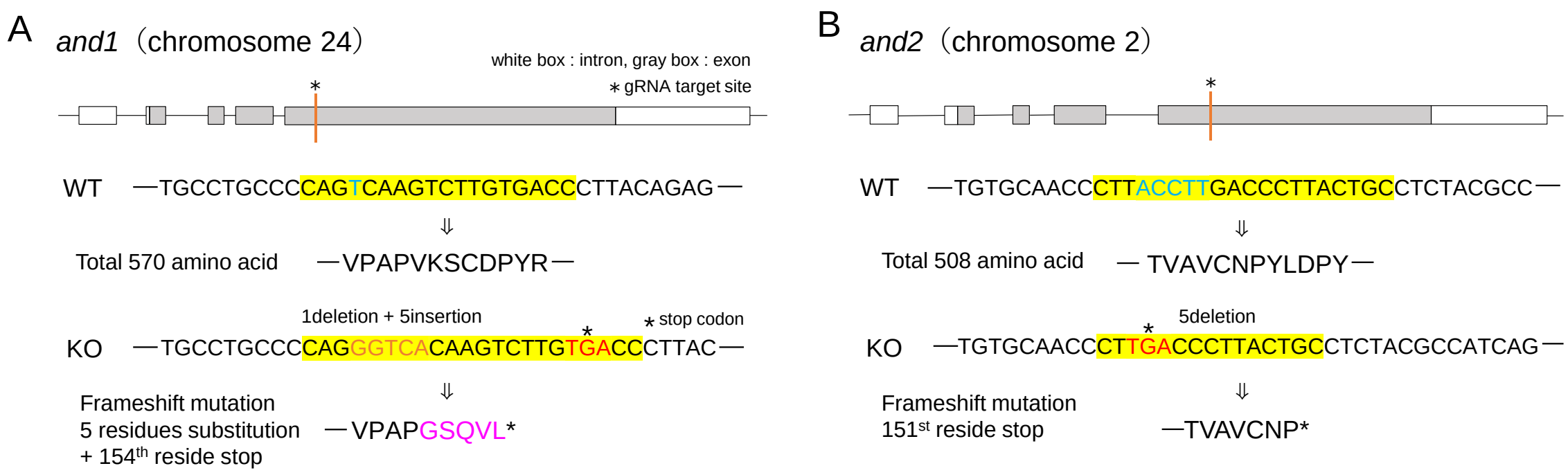

Figure S1. Frame-shift mutations indicate that each *and1/2* KO sequence lacks more than 70% of the amino acids in the respective WT-encoded proteins, related to Figure 1
(A, B) Locus information for the wild-type (WT) vs. knockout (KO) alleles of *and1* / *and2*. These annotations were referenced from the UCSC Genome Browser (Zebrafish May 2017 release (GRCz11/danRer11), *and1*_mRNA (chr24:26279503-26283359, NM_001197254) / *and2*_mRNA (chr2:31763202-31767652, NM_001111190). Yellow box: gRNA target region; blue text: deletion site; orange text: insertion site; magenta text: substituted amino acid.

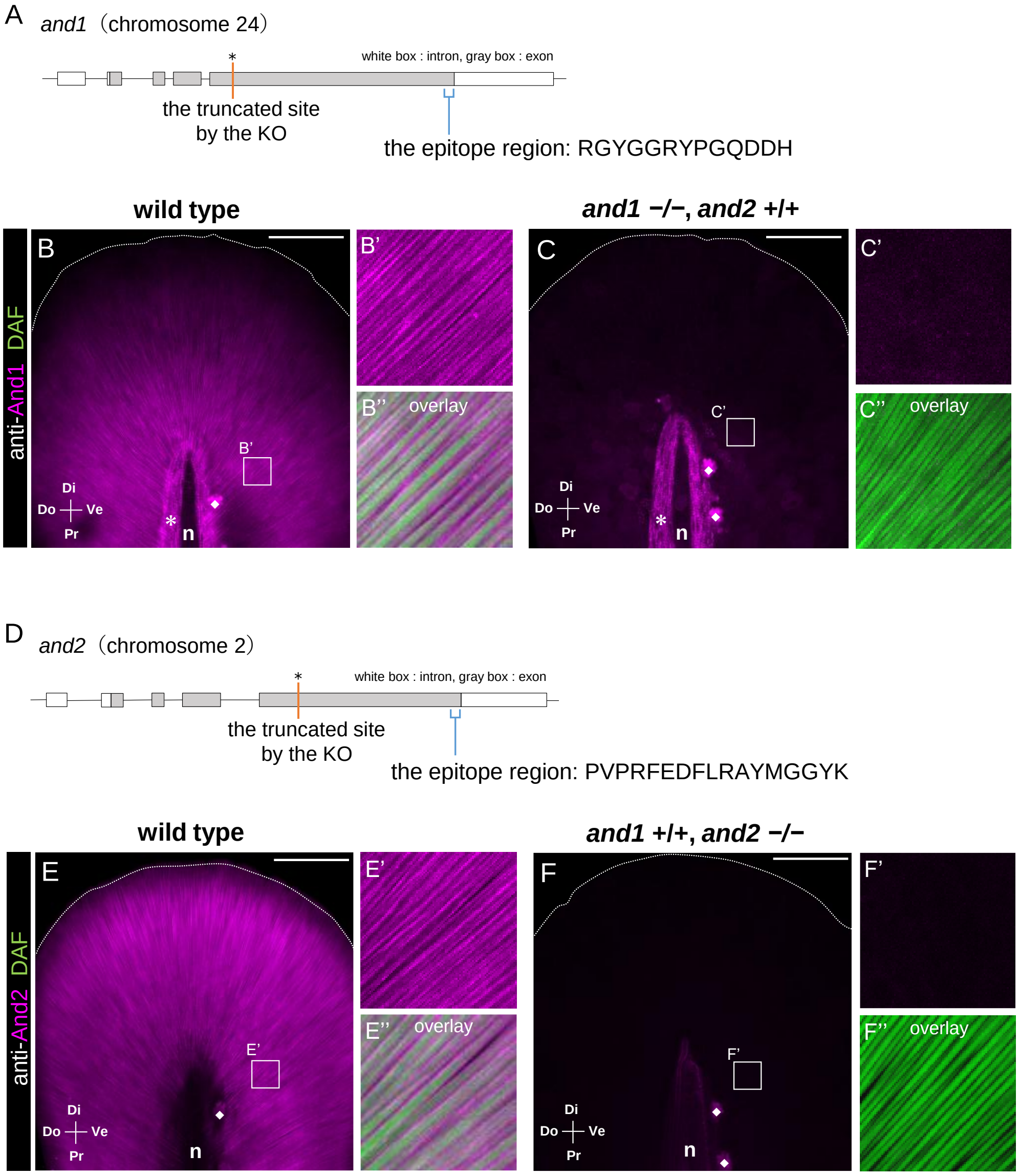

Figure S2. Both the *and1* and *and2* knockout alleles disrupt expression of the functional WT protein, related to Figure 1 (A) Epitope information for the anti-And1 antibody. (B, C) Confocal images (MIP) of the caudal fin fold in wild-type and *and1* knockout zebrafish at 5 dpf. Whole-mount tissues were stained with the anti-And1 antibody (magenta) and DAF (green). Enlarged views of the white-boxed region are shown in the right panels (B’, C’). (D) Epitope information for the anti-And2 antibody. (E, F) Confocal images (MIP) of the caudal fin fold in wild-type and *and2* knockout zebrafish at 5 dpf. Whole-mount tissues were stained with the anti-And2 antibody (magenta) and DAF (green). Enlarged views of the white-boxed region are shown in the right panels (E’, F’). White symbols indicate nonspecific signals derived from polyclonal antibodies. White diamonds: neuromasts; white asterisks: myofibers. Pr: proximal; Di: distal; Do: dorsal; Ve: ventral; n: notochord; MIP: maximum intensity projection. Scale bars: 100 μm.

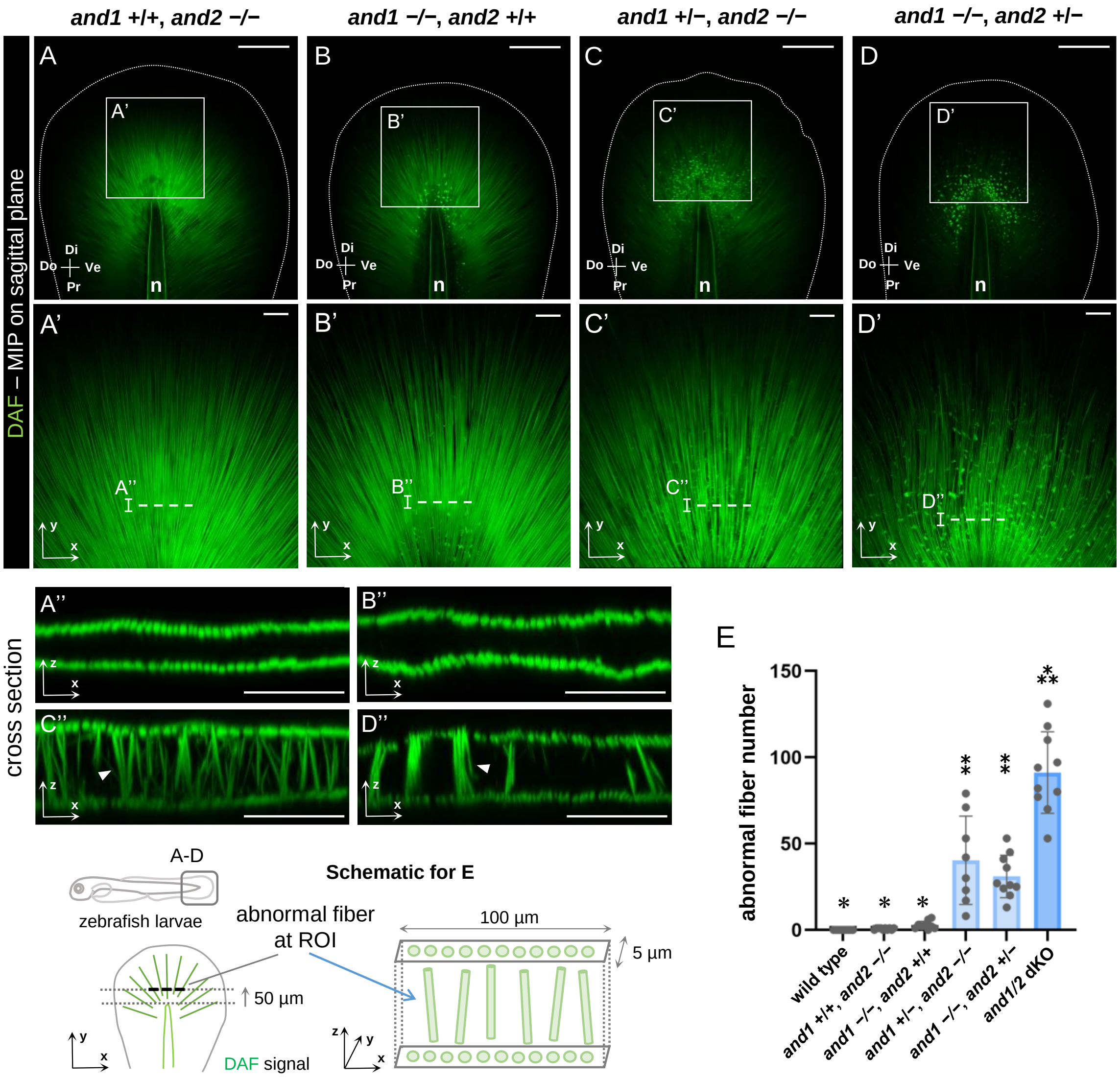

Figure S3. Collagen fiber orientation in the caudal fin folds of zebrafish from different *and1/and2* mutant genotypes, related to Figure 1 (A-D'') Confocal images of caudal fin folds from each *and1* and *and2* knockout genotype at 5 days post-fertilization (dpf). Collagen fibers were fluorescently labeled (green) by whole-mount DAF staining. Magnified views of the white-boxed regions are shown in the lower panels. Cross-sectional views corresponding to panels A'-D' are also shown below (MIP, 5-µm stack, the region 50 µm from the notochord end). White dotted lines: fin outlines; white arrowheads: abnormal vertical fibers. Scale bars: 100 µm (A-D), 20 µm (A'-D''). (E) Quantification of the number of abnormal vertical fibers (the region 50 µm from the notochord end). Data are presented as mean ± SD. Tukey-Kramer method; different symbols indicate $p<0.05$. N (left to right: WT to dKO) = 10, 10, 9, 8, 10, 10.

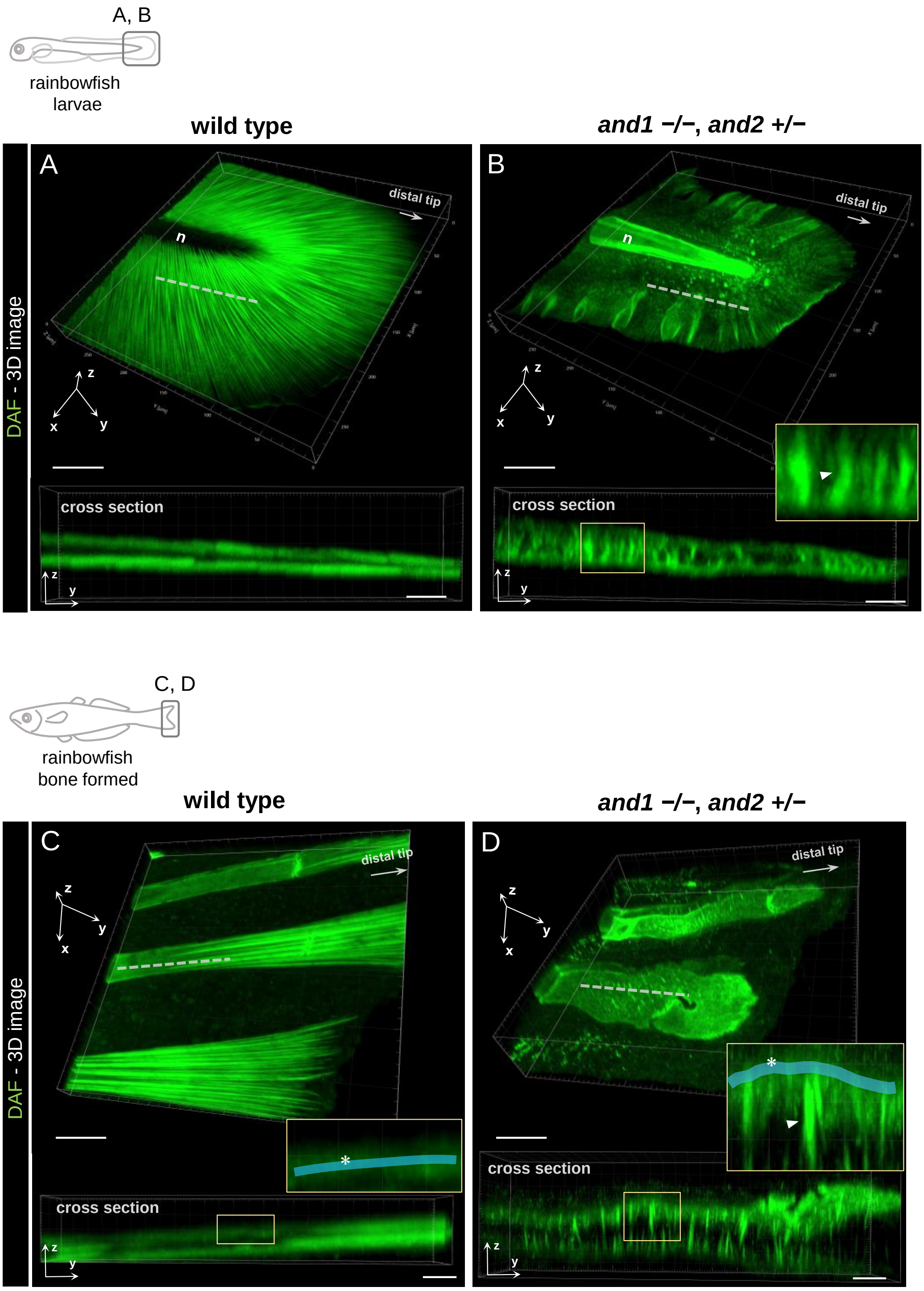

Figure S4. Abnormal vertical fibers are distributed in the caudal fin of *and1/2* double mutant rainbowfish, resembling that in the *and1/2* dKO zebrafish line, related to Figure 1 and Figure 3 (A, B) Confocal images of the caudal fin fold in wild-type and *and1/2* double mutant rainbowfish at 7 dpf. Collagen fibers were fluorescently labeled (green) by whole-mount DAF staining. Cross-sectional views at the white dotted lines are shown in the lower panels. (C, D) Confocal images of the caudal fin tip in wild-type and *and1/2* double mutant rainbowfish at DF-st4. Collagen fibers were fluorescently labeled (green) by

whole-mount DAF staining. Cross-sectional views at the white dotted lines are shown in the lower panels. White arrowheads: abnormally oriented collagen structures; white asterisks with translucent blue lines: collagen matrices within fin bones. Scale bars: 50 μm (3D views, A-D), 10 μm (cross-sectional views, A-D).

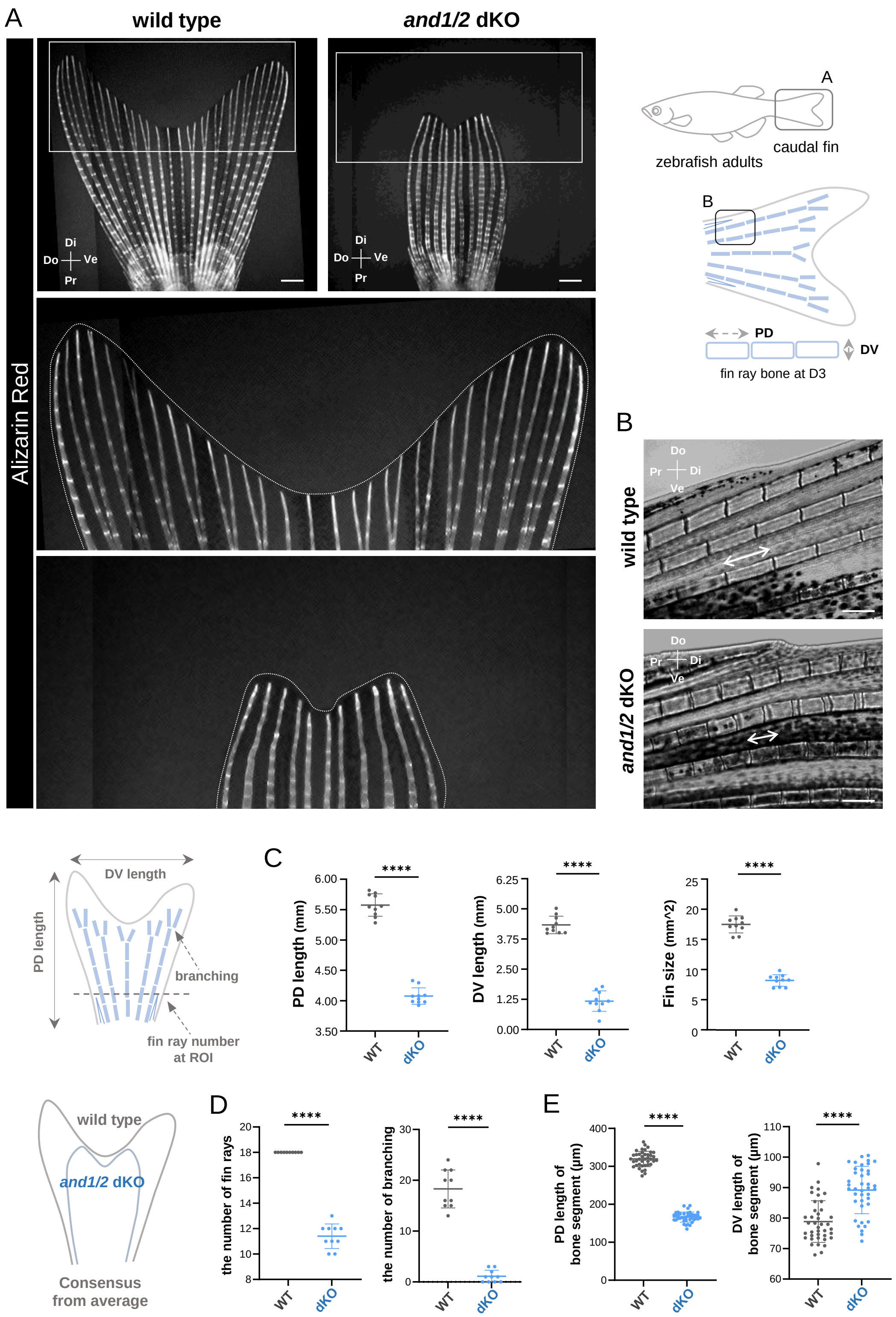

Figure S5. ***and1/2*** **dKO zebrafish exhibit morphological defects in the caudal fin, related to Figure 3** (A) Fluorescence images of the caudal fin stained with Alizarin Red in wild-type and *and1/2* dKO zebrafish with standard length (SL) = 2.1 ± 0.1 cm (adults over 6 months of age). White dotted lines: fin outlines. Scale bars: 500 μm. (B) Magnified images of the fin bones in wild-type and *and1/2* dKO. Double-headed arrows indicate the single-segment structure of fin bones. Scale bars: 200 μm. (C-E) Quantification of fin morphology. Data are

presented as mean ± SD. Welch's t-test: **** $p < 0.0001$. WT vs. dKO. Fin skeleton: N = 10 per group; Bone segments: N = 40 per group (10 fishes per group).

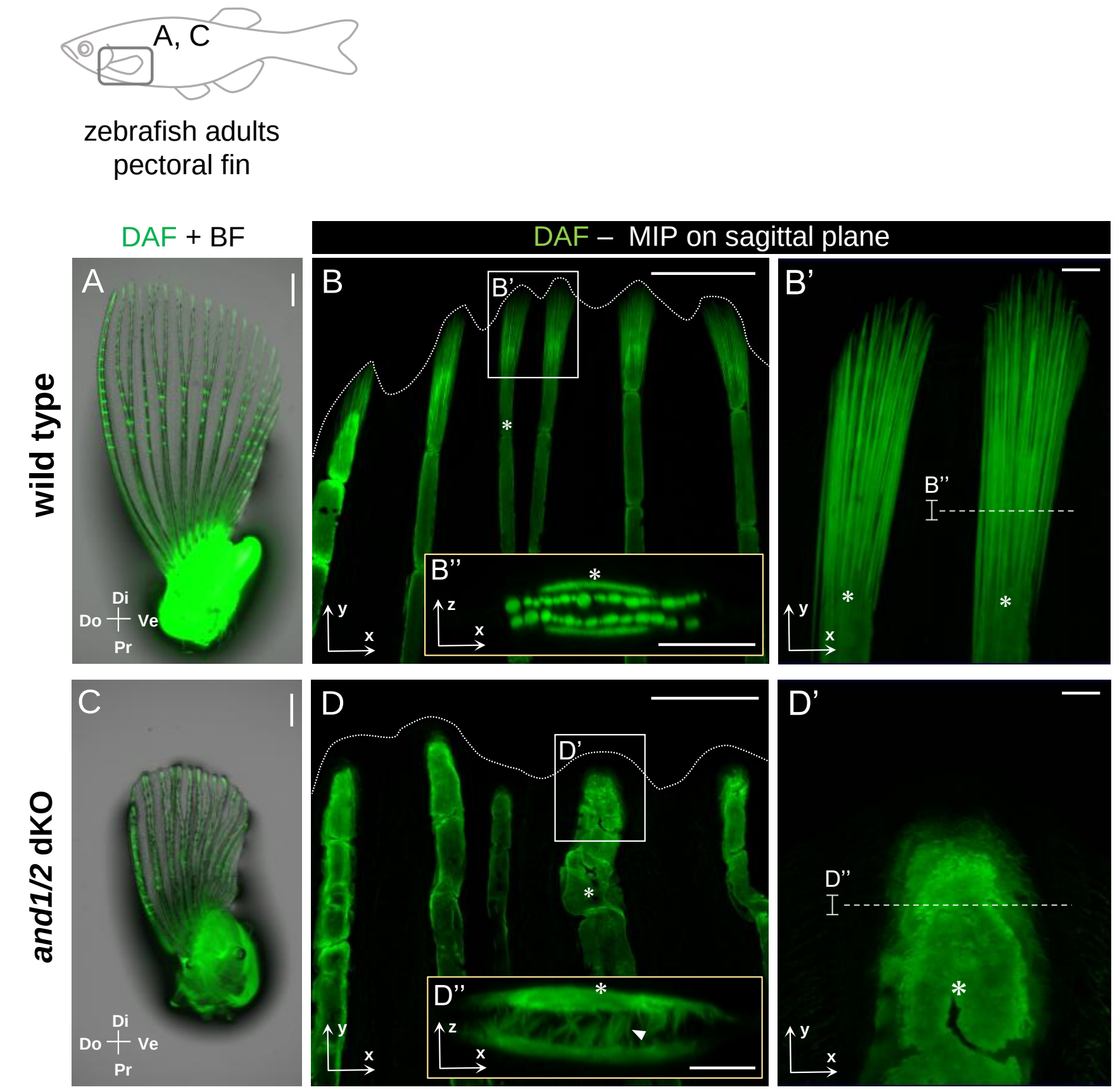

Figure S6. *and1/2* dKO zebrafish display same abnormality in the pectoral fin as in the caudal fin, related to Figure 3 (A, C) Fluorescence images of the pectoral fin stained with DAF in wild-type and *and1/2* dKO zebrafish with SL = 2.1 ± 0.1 cm (adults over 6 months of age). Scale bars: 500 μm. (B-B'', D-D'') Confocal images of the pectoral fin tip in wild-type and *and1/2* dKO (MIP, 10-μm stack (B'', D'')). White dotted lines: fin outlines; white asterisks: collagen matrices within fin bones; white arrowhead: abnormally oriented collagen structures. Scale bars: 200 μm (B, D), 20 μm (B'-B'', D'-D'').
